# Error-Related Negativity-Based Robot-Assisted Stroke Rehabilitation System: Design and Proof-of-Concept

**DOI:** 10.3389/fnbot.2022.837119

**Published:** 2022-04-25

**Authors:** Akshay Kumar, Lin Gao, Jiaming Li, Jiaxin Ma, Jianming Fu, Xudong Gu, Seedahmed S. Mahmoud, Qiang Fang

**Affiliations:** ^1^Department of Biomedical Engineering, College of Engineering, Shantou University, Shantou, China; ^2^OMRON SINIC X Corporation, Tokyo, Japan; ^3^2nd Hospital of Jiaxing, Jiaxing, China

**Keywords:** assist-as-needed (AAN), brain-computer interface (BCI), error-related potentials (ErrP), robot-therapy, single-trial classification, stroke rehabilitation, Training-ErrPs

## Abstract

Conventional rehabilitation systems typically execute a fixed set of programs that most motor-impaired stroke patients undergo. In these systems, the brain, which is embodied in the body, is often left out. Including the brains of stroke patients in the control loop of a rehabilitation system can be worthwhile as the system can be tailored to each participant and, thus, be more effective. Here, we propose a novel brain-computer interface (BCI)-based robot-assisted stroke rehabilitation system (RASRS), which takes inputs from the patient's intrinsic feedback mechanism to adapt the assistance level of the RASRS. The proposed system will utilize the patients' consciousness about their performance decoded through their error-related negativity signals. As a proof-of-concept, we experimented on 12 healthy people in which we recorded their electroencephalogram (EEG) signals while performing a standard rehabilitation exercise. We set the performance requirements beforehand and observed participants' neural responses when they failed/met the set requirements and found a statistically significant (*p* < 0.05) difference in their neural responses in the two conditions. The feasibility of the proposed BCI-based RASRS was demonstrated through a use-case description with a timing diagram and meeting the crucial requirements for developing the proposed rehabilitation system. The use of a patient's intrinsic feedback mechanism will have significant implications for the development of human-in-the-loop stroke rehabilitation systems.

## Introduction

Recently, stroke has become the second leading cause of death and the third leading cause of disability worldwide (Feigin et al., 2017). In 2013 alone, there were 10.3 million new stroke incidents, and year after year, the stroke incident rate is rapidly increasing (Feigin et al., 2017). Depending upon which part of the brain has suffered damages due to stroke, the stroke survivors may suffer from various types of functional impairment, such as cognitive impairment, motor impairment, speech, and language impairment, and even death. When the damage due to stroke is in the brain's motor cortex, the patients with stroke lose their ability to voluntarily move their limbs (Kumar et al., 2019a). The limb movement, especially the upper-limb movement, plays a significant role in carrying out *activities of daily living* (ADL) and without it, leading a life independently becomes difficult. Thus, regaining control of the upper limb is significant for patients with quadriplegia (Anderson, 2004).

In hospitals, motor-impaired people undergo post-stroke rehabilitation procedures, in which they perform physical rehabilitation exercises using their impaired limbs. For instance, patients having problems controlling their shoulder muscles can perform shoulder flexion-extension and horizontal flexion-extension movement as part of their post-stroke rehabilitation program. These programs stimulate the brain's process of neurological changes and have been shown to help motor-impaired stroke patients regain the lost motor functionality (Warraich and Kleim, 2010; Keci et al., 2019). In other words, a higher number of stimuli to the damaged part of the brain, by performing the rehabilitation exercises repeatedly, can lead to a greater amount of recovery (Lohse et al., 2014; De Sousa et al., 2018; Keci et al., 2019). This notion is supported by many studies that link the amount and rate of recovery from motor impairment with prolonged, repetitive, and active participation in rehabilitation programs (Banala et al., 2009; Warraich and Kleim, 2010; Lohse et al., 2014; De Sousa et al., 2018).

Now the question remains, how can we ensure active and prolonged participation of a stroke patient in a rehabilitation program? A stroke patient, especially in the acute stage, can have weak muscle strength and severe muscle pain and can find it challenging to participate in a prolonged and active rehabilitation session. Nevertheless, Shirzad and Van Der Loos (2016) showed that participants continued longer in robot-assisted training sessions than the control group on giving desirable difficulty levels to participants in a reaching motion task. They reported that desirable difficulty made the sessions less frustrating and more engaging, which improved participants' training experience. However, in practice, the rehabilitation procedures with manual interventions, which require rehabilitation therapists and doctors to prescribe rehabilitation exercises to patients based on their current motor impairment level, are prevalently used. As stroke incidents are increasing year after year (Feigin et al., 2017), it is becoming increasingly difficult for rehabilitation doctors and therapists to observe individual patients on a repeatable basis and continually adapt their rehabilitation strategy. This is becoming more common in low- and medium-income countries, where stroke patients are left to follow fixed rehabilitation programs without much adaptation.

Nevertheless, the need of the hour is to use rehabilitation methods that can automatically infer the motor impairment level of stroke patients, adapt the rehabilitation exercise difficulty level, and can provide external assistance to the patient in performing the rehabilitation exercise as needed [i.e., *assist-as-needed* (AAN) (Banala et al., 2009; Basteris et al., 2014)] to ensure active participation of the patient. Recently, several studies have proposed robot-assisted rehabilitation strategies for developing AAN rehabilitation programs, in which external assistance can be provided to the participant as needed (Colombo et al., 2007; Banala et al., 2009; Song et al., 2013). For instance, Song et al. (2013) proposed an EMG-controlled wrist robot in which the assistance level given by the robot to perform the rehabilitation movement was made proportional to the EMG activity of flexor muscle and extensor muscle of the affected upper limb. Colombo et al. (2007) demonstrated a force/torque transducer-based robot-assisted rehabilitation system in which external assistance was made proportional to the force exerted by the patient on the transducer mounted on a robotic handle. Liu et al. (2017) demonstrated a brain-actuated lower-limb exoskeleton, which utilized sensorimotor rhythms (SMR) and movement-related cortical potentials as control signals and partially implemented the AAN approach for lower-limb gait training. Recently, the end-effector-based InMotionRobots™ (Bionik Laboratories Corp., Toronto, ON, Canada) is also becoming popular for their interactive upper-limb rehabilitation programs, among other modalities (Basteris et al., 2014). However, in clinical settings, the RASRSs have failed to impress clinicians (Hatem et al., 2016; Rodgers et al., 2019). Recently, a large-scale study conducted by Rodgers et al. (2019) concluded that robot-assisted rehabilitation training delivered using MIT-Manus robotic gym system does not show any additional gain in upper-limb function when compared with the enhanced upper limb therapy (EULT) and the usual care for stroke patients, when delivered at the same frequency and duration (Rodgers et al., 2017). Now, if we trust our fundamental knowledge behind stroke recovery that prolong, repetitive, and active participation in the rehabilitation program results in a greater rate and amount of recovery from motor impairment (Banala et al., 2009; Warraich and Kleim, 2010; Lohse et al., 2014; De Sousa et al., 2018), then it might be said that existing RASRSs are failing to ensure active participation of stroke patients in the rehabilitation program and adapt to patients' desired level of difficulty, which results in unexpected amount and rate of recovery (Hatem et al., 2016; Rodgers et al., 2019).

Consider a practical situation in which a stroke patient is told to put maximum physical effort to perform a rehabilitation exercise while using a RASRS; plainly, the patient can evaluate if the external assistance provided to him/her is optimum or is more/less than needed. If such information can be extracted from the patient's brain, it can help in adapting the external assistance. A strategy with similar essence was presented by Rotermund et al. (2006), in which they proposed the use of a hypothetical brain signal, which reflects the user's affective evaluation of intended movement and the movement executed by a neuro-prosthetic. They proposed to use the hypothetical brain signal to minimize the difference between the intended movement and the executed movement. An error-related potential (ErrP) signal can be used in place of this hypothetical brain signal (Chavarriaga and Millán, 2010; Salazar-Gomez et al., 2017; Kumar et al., 2019b).

The ErrP signal is an event-related potential (ERP) signal which elicits in the human brain, be it healthy people or motor-impaired stroke patients (Chavarriaga and Millán, 2010; Kumar et al., 2019b). It is generally described as the difference of the error-related negativity (ERN) signal, which elicits in the brain following the perception of an error, and the correct-related activity (CRA), which elicits following the perception of correctness (Chavarriaga and Millán, 2010; Kumar et al., 2019b). The ErrP signal has been observed to be elicited in various task situations (e.g., while observing a moving cursor, interacting with a robot, among others), and a wide range of ErrP-based BCI applications have been developed for motor-impaired people (Chavarriaga and Millán, 2010; Salazar-Gomez et al., 2017; Yazmir and Reiner, 2017; Kumar et al., 2019b). Recently, an ErrP signal was observed to elicit while stroke patients performed physical rehabilitation exercises (named Training-ErrP hereafter) (Kumar et al., 2019a). According to Kumar et al. (2019a), the ERN associated with the Training-ErrP signal is elicited in the brain of a stroke patient if the patient fails to complete a rehabilitation exercise in a given time. The ERN signals represent the human brain's intrinsic feedback mechanism. These signals can elicit unconsciously in the brain on a real-time basis and do not require any training, unlike other EEG signals, such as SMR (Chavarriaga and Millán, 2010; Liu et al., 2017; Salazar-Gomez et al., 2017). These characteristics of the ERN associated with the Training-ErrP signal can potentially facilitate the development of a RASRS, which can adapt the robotic assistance given to the stroke patient, for every exercise trial. However, in literature, no discussion has been made on Training-ErrP-based RASRSs, so far.

Therefore, in this paper, we proposed a novel ERN-based RASRS. The proposed ERN-RASRS uses an off-the-shelf rehabilitation robot to provide external assistance to patients with a stroke to perform rehabilitation exercises. The ERN signal forms the basis of increasing/decreasing the external assistance level on a trial-by-trial basis. Considering the importance of ERN signal in ERN-RASRS, as a proof-of-concept, we experimented on 12 healthy people, wherein we recorded their EEG signals while they were performing a standard rehabilitation exercise and analyzed their neural response when they failed to complete the rehabilitation exercise in a given time against when they succeeded. We also evaluated the single-trial classification performance of the ERN signals against its counterpart, i.e., CRA, and against the background EEG activity asynchronously.

It is to be noted that this study, in particular, demonstrates the design and the proof-of-concept of the proposed ERN-based RASRS. However, the ERN-based RASRS would be fabricated and evaluated against the current state-of-the-art approaches in our upcoming studies in the near future. Nevertheless, the contributions made in this research article have initiated the development of the ERN-based RASRS. Section ERN-based Robot-Assisted Stroke Rehabilitation System of the paper discusses the design and a use-case description of the proposed ERN-RASRS. Section Materials and Methods and onwards present the proof-of-concept of the system.

## ERN-Based Robot-Assisted Stroke Rehabilitation System

### Fundamental Operation

**Figure 1:**
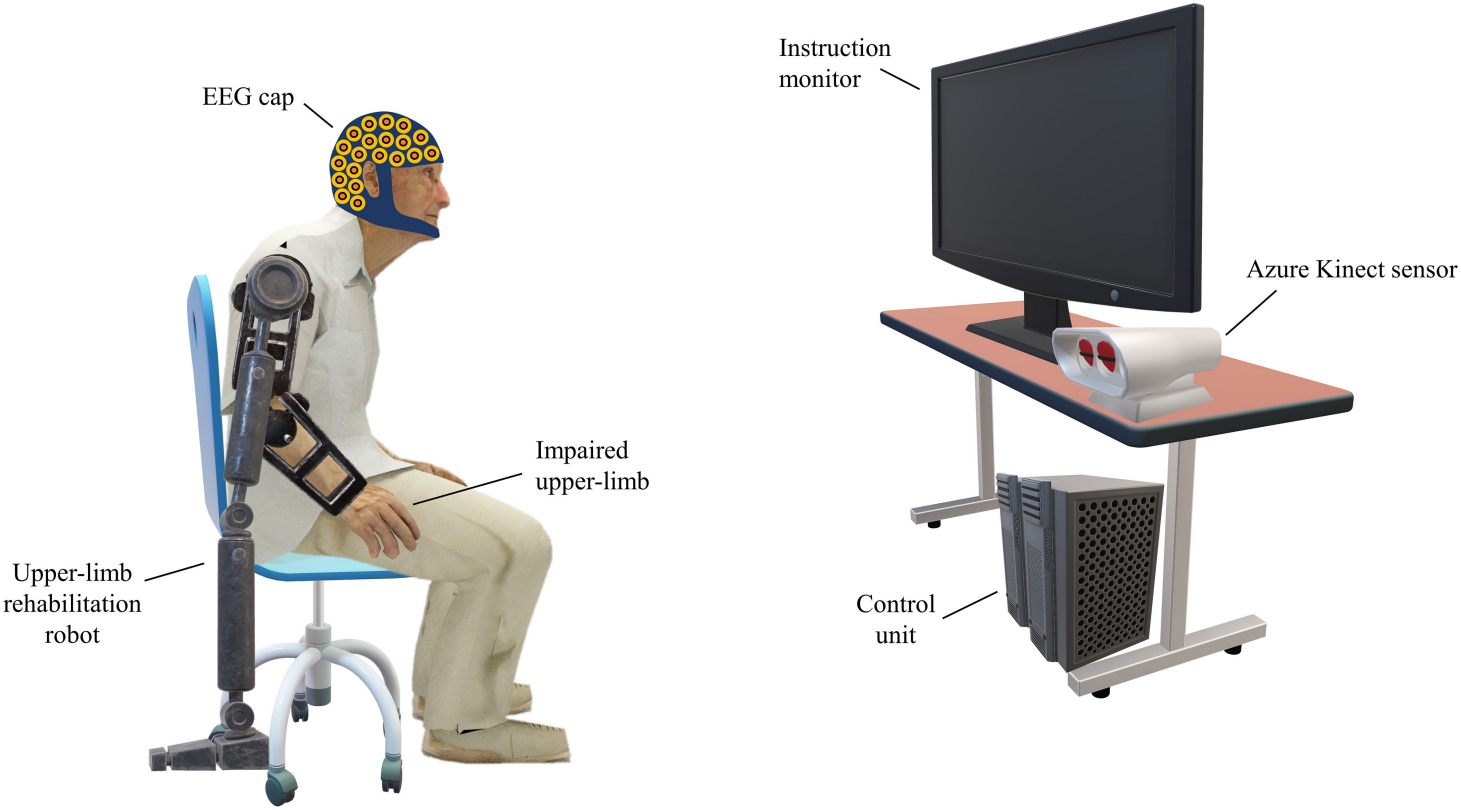
Setup of the event-related negativity (ERN)-based robot-assisted stroke rehabilitation system (RASRS). The rehabilitation robot would assist the patient in performing the rehabilitation exercise as required. The instruction monitor would guide the patient in performing the rehabilitation exercise by showing relevant cues on the screen and would also show the real-time feedback of the movement of the impaired limb with the help of the Azure Kinect sensor, so that the patient can know how much exercise has been performed and how much it is left. The electroencephalogram (EEG) sensors record the EEG activity from the patient's brain. The control unit would process the EEG data, the Kinect sensor data, give inputs to the instruction monitor, and signals the rehabilitation robot to increase/decrease the robotic assistance.

Figure 1 depicts the setup of the ERN-based RASRS and its various constituting parts. The instruction monitor would guide the stroke patient through the rehabilitation training procedure by giving instructions and cues on rehabilitation exercises. While the patient is performing the rehabilitation exercise, the Azure Kinect sensor (Microsoft Corporation, Redmond, WA, US) (Azure Kinect) would track the patient's limb movement. Azure Kinect sensor, together with the instruction monitor, would show the real-time feedback of the movement of the patient's impaired limb so that the patient can know how much exercise he/she has completed, and the position of his/her limb relative to the target of the current exercise. Here, the target describes the level of exercise difficulty; for instance, for a stroke patient with upper-limb impairment performing a shoulder flexion movement, the target would define up to what height the patient has to reach.

The rehabilitation robot can be an end-effector or an exoskeleton, whose main task would be to assist stroke patients in performing rehabilitation exercises. Now an exercise's target is defined; the patient, with no external assistance or fixed minimum external assistance from the rehabilitation robot (discussed in detail in the following subsection), would attempt to reach the target. Simultaneously, the EEG signals would be recorded from the patient's brain. If the patient fails to reach the defined target of the exercise, an ERN signal associated with the Training-ErrP signal will elicit in the patient's brain, which, from the patient perspective, would indicate that he/she has failed to complete the rehabilitation movement. Thus, on the ERN signal detection, the external assistance would be increased/triggered so that the patient can complete the rest of the rehabilitation exercise. In the trials of subsequent sessions, the exercise's difficulty level can be decreased through the exercise's target, or the fixed minimum assistance level can be increased, to adapt the rehabilitation system as per the patient's current motor impairment level.

The control unit will process the EEG signals and the Kinect sensor's data in real-time, increase/decrease the assistance level of the rehabilitation robot, and adapt the exercise target level for every exercise trial, based on the detection of ERN signal and the past performances of the patient in performing rehabilitation exercises (tracked through the Kinect sensor).

### Use-Case Description Using Timing Diagrams

**Figure 2:**
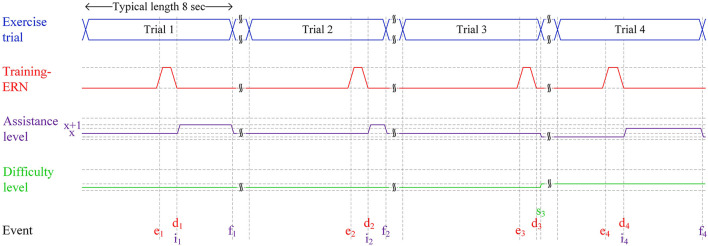
Four typical trials of the ERN-RASRS illustrated using a timing diagram. The durations in the figure are not scaled. Event *e* marks the time when the ERN associated with the Training-ErrP signal elicits. Event *d* marks the time when the ERN signal is detected. *x* shows the minimum level of assistance provided by the rehabilitation robot throughout the exercise trial. The *x*+*1* shows the level of assistance provided by the rehabilitation robot on the detection of the ERN signal. Event *i* marks the time when robotic assistance is increased to level *x*+*1*. Event *f* marks the time when the current trial finishes and the robotic assistance is decreased to level *x*. Event *s* marks an exercise trial in which almost the whole exercise is performed only with the minimum level of robotic assistance (i.e., *x*); thus, the difficulty level of the exercise is increased, and the minimum level of robotic assistance (i.e., *x*) is decreased.

Figure 2 depicts how a typical set of four trials of a patient at various levels of motor impairment during his/her rehabilitation journey would unfold in an ERN-RASRS. At the start, the rehabilitation robot would maintain a fixed minimum assistance level (*x* in Figure 2) to support a stroke patient who is at the initial stage of motor recovery. Typically, such a patient has little to no voluntary movement in his/her impaired limb and requires continuous external support to perform rehabilitation exercises. The minimum assistance level can be gradually decreased as the patient gains voluntary limb movement.

In Trial 1, despite the external assistance, the stroke patient perceived that he/she has failed in performing the rehabilitation exercise due to motor impairment and cannot reach the set target; thus, an ERN signal associated with Training-ErrP is elicited (*e*_1_ in Figure 2). As the EEG signals are being processed in real-time, the elicited ERN signal gets detected at *d*_1_ in Figure 2. In response to this, the external robotic assistance level is increased to *x*+*1* level (*i*_1_ in Figure 2), which helps the patient complete the rest of the exercise. The assistance level is decreased back to level *x* on the completion of Trial 1 (*f*_1_ in Figure 2), i.e., the patient has reached the target with the help of an increased assistance level.

A similar set of events unfolds in Trial 2; however, as the patient is getting better at performing the rehabilitation exercise, the duration for which the patient requires a higher level of external assistance (i.e., *x*+*1*) is decreased. In Trial 3, the patient further gets better in performing the rehabilitation exercise and performs almost the whole exercise with the minimum level of robotic assistance (i.e., *x*). The extent of the exercise completed is additionally verified with the Azure Kinect sensor's data. As a result, the ERN signal also elicits near the very end of the trial (*e*_3_ in Figure 2). It shows that the stroke patient's motor ability has nearly recovered to the level that the patient can complete the given rehabilitation exercise with the current fixed minimum level of external assistance (*x* in Figure 2). It is to be clarified that a patient would require multiple rehabilitation sessions before experiencing a noticeable gain in motor skills, although the recent discussion might give a perception that the patient is regaining motor skills in subsequent trials.

To ensure maximum effort from the patient and keep the rehabilitation program continually challenging, the rehabilitation program must be adapted to the current motor ability of the stroke patient. Two strategies can be employed in this scenario: the fixed minimum level of external assistance can be decreased (*x* in Figure 2), and/or the exercise's difficulty level can be increased (i.e., by modifying the target level). In Trial 4, both strategies are utilized. The fixed minimum level of external assistance is decreased, and the difficulty level of the exercise is also slightly increased (*s*_3_ in Figure 2). In response to this, the patient again failed in completing the exercise trial, and an ERN signal is elicited (*e*_4_ in Figure 2) and consequently, a higher level of external assistance is given (*i*_4_ in Figure 2), so that the patient can complete the rest of the exercise trial.

Hence, the functioning of the ERN-RASRS has been demonstrated in which the robotic assistance level and the exercise difficulty level are adapted on a trial-by-trial basis as per the current motor-impairment level of the patient. This can keep the rehabilitation program continually challenging and ensure maximum efforts from patients, along with their active participation. The upcoming sections present the system's proof-of-concept by studying the ERN signals elicited while performing a physical rehabilitation movement and its single-trial classification.

## Materials and Methods

### Participants

In the experiment, twelve healthy people participated [7 females and 5 males, mean (M) age: 22.8 ± 3.2 years]. None of the participants had a BCI experience. The experiment was conducted at Shantou University, China. All participants voluntarily participated in the experiment and signed a written informed consent before the experiment started. The study was approved by the ethics committees of the 2nd Hospital of Jiaxing and the First Affiliated Hospital of Shantou University Medical College, China. The experiment was conducted by the declaration of Helsinki. One of the participants' data were not included in the analyses as the events information for his experiment went unrecorded due to a technical glitch in the EEG recording software.

### Experiment Protocol

**Figure 3:**
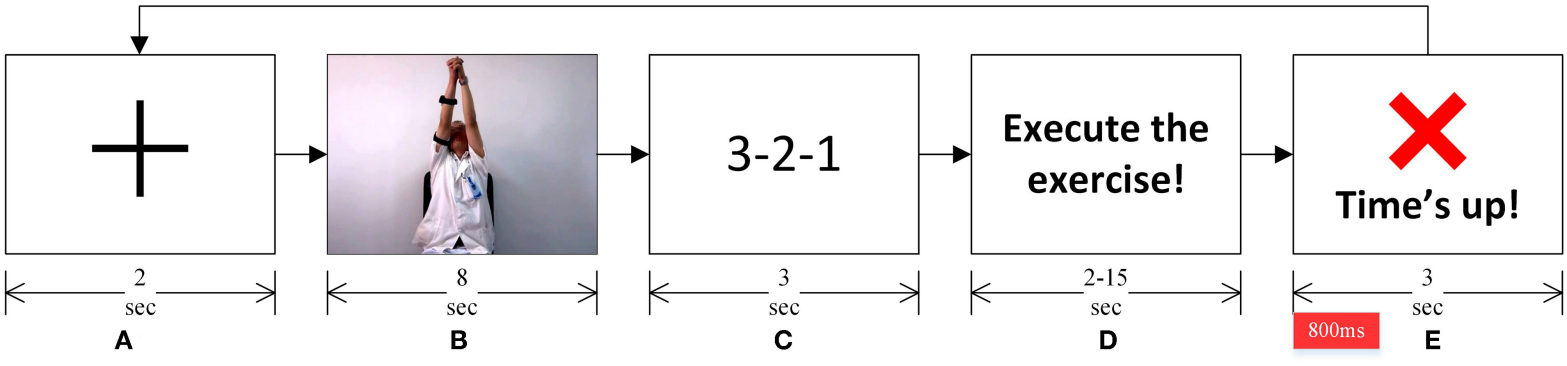
**(A)** A fixation cross marked the start of a trial. **(B)** A pre-recorded video showed the rehabilitation exercise to be performed. **(C)** A 3-2-1 timer. **(D)** Instruction to the participants to start performing the rehabilitation exercise depicted in the video. **(E)** Participants were asked to complete the rehabilitation exercise before this “Time's up!” screen appeared. This marked the completion of one exercise trial, and subsequently, the next trial started with the fixation cross. The epoch of interest for EEG analyses is marked in red on the timeline, immediately after the “Time's up!” screen appeared.

Participants sat on a chair facing a LED monitor with 1,920 × 1,080 resolution at a 60 Hz refresh rate. The experiment required participants to perform a standard Bobath's rehabilitation exercise involving shoulder flexion-extension while adjoining both hands. The experiment was conducted in a synchronous format, and the experiment instructions and cues were delivered using the LED monitor, in order, as shown in Figure 3. A Pre-recorded video of a rehabilitation therapist, obtained from the 2nd Hospital of Jiaxing, China, was used to describe the know-how of the rehabilitation exercise.

As shown in Figure 3A, a fixation cross marked the start of an experiment trial. The rehabilitation exercise video followed the fixation cross. Participants were instructed to observe the exercise movement in the video (see Figure 3B). A 3-2-1 timer followed the video (see Figure 3C). Participants were asked to perform the rehabilitation exercise (as depicted in the rehabilitation video) once the timer finishes and the screen, as shown in Figure 3D, appeared. Participants were told that they must complete performing the exercise, with the same range of motion and speed as depicted in the exercise video (Figure 3B), before the “Time's up!” screen (see Figure 3E) appeared. They were also asked to evaluate in their head if they completed the exercise once the “Time's up!” screen appeared. On the onset of the “Time's up!” screen, a beep sound was also played for about 30 ms to notify participants that the time duration given to complete the rehabilitation exercise has expired. This marked the completion of one exercise trial, and each participant participated in forty-eight such trials, where they performed the same shoulder flexion-extension exercise.

The duration given to the participants to complete the rehabilitation exercise varied from 2 to 15 s pseudo-randomly. Specifically, 2–4 s were given in 18 of the 48 trials, 9–10 s were given in four of the 48 trials, and 15 seconds were given in 26 of the 48 trials, to all participants to complete the rehabilitation exercise. These time-durations, together with the instructions given to participants on how to perform the exercise (described in the preceding paragraph), ensured that participants failed to complete the rehabilitation movement in the 2–4 s trials (named ERN trials hereafter) and successfully completed it in 15 s trials (named CRA trials hereafter). An exercise trial was marked as successful when a participant started performing the exercise on the onset of the screen as in Figure 3D, executed the exercise as shown in the exercise video, and his/her arms come to rest before the onset of “Time's up!” screen (see Figure 3E). All ambiguous trials were removed before the analyses; about 6% of the trials were removed. The 9–10 s trials were used to add uncertainty in the experiment so that participants could not foretell the given time durations, and these trials were excluded from the analyses. A preliminary experiment was also carried out on three healthy people (different from the 12 participants mentioned earlier) to confirm that the time length given in a trial to perform the exercise cannot be anticipated. After the preliminary experiment, an interview-based analysis was also conducted on the three participants to understand and ensure that they did perceive an erroneous action in the ERN trials for not completing the exercise in the given time. Such perception is essential to evoke ErrP related neural response (Taylor et al., 2007; Salazar-Gomez et al., 2017; Yazmir and Reiner, 2017).

The experiment procedure was explained to the participants, and sufficient time was given for practice before the experiment started. The experiment's visual stimuli were designed and delivered using the Presentation software (version 21.1, Neurobehavioral Systems Inc., California).

### EEG Data Acquisition

Thirty-two channels of EEG (i.e., FP1, FP2, AF3, AF4, F7, F3, Fz, F4, F8, FC5, FC1, FC2, FC6, T7, C3, Cz, C4, T8, CP5, CP1, CP2, CP6, P7, P3, Pz, P4, P8, PO7, PO3, PO4, PO8, and Oz) were recorded using g.Nautilus (g.tec GmbH, Austria) wireless EEG acquisition system, as per the international 10–20 system. Eight-pin g.SAHARA dry-active EEG electrodes mounted on a g.GAMMAcap were used for the EEG recording. All channels were referenced to the right mastoid and grounded to the AFz location. The EEG data were acquired at a 500 Hz sampling rate with a 50 Hz notch filter.

### Data Pre-Processing and Analyses

The recorded raw EEG data were pre-processed offline before making any analysis. The MATLAB-based custom scripts and EEGLAB toolbox (Delorme and Makeig, 2004) were used for the pre-processing. The data were band-pass filtered in the 0.1–50 Hz range using a zero-phase shift FIR filter. Afterward, the data were manually inspected to remove any transients and artifactual data segments. Subsequently, a combination of Automatic channel rejection (an EEGLAB plug-in) and manual inspection was used to remove any artifactual channels from the dataset. Next, the data were downsampled to 250 Hz, and subsequently, the Independent component analysis (Delorme and Makeig, 2004) was used to remove artifactual components left in the data, such as ocular, cardiac, and muscle artifacts. A combination of ICLabel (an EEGLAB plug-in) and visual inspection was used to remove the artifactual components. The continuous data were segmented into epochs ranging from −100 to 800 ms relative to the onset of Figure 3E, and the individual epochs' means were subtracted from the epochs themselves. Further, the removed data channels were interpolated. Finally, the events associated with ERN trials and CRA trials were separated. On average, about 41 trials per participant were included in the analyses, out of which about 16 were ERN trials, and 25 were CRA trials. These trials were used for the single-trial classification of ERN and CRA signals (discussed in detail in Section Single-Trial Classification).

For every participant, the average of the ERN and CRA trials was calculated. Subsequently, grand-average ERN and CRA were calculated by taking the average of the individual averaged ERN and CRA waveforms. The difference of the averaged ERN and CRA waveforms was calculated for every participant to calculate their averaged Training-ErrP waveforms. The grand-average Training-ErrP waveform was also calculated by averaging the Training-ErrP waveform of every participant. The time-domain EEG responses suffer from reference-electrode bias; therefore, topographical scalp maps of the grand-average ERN and CRA neural activities were also calculated to obtain reference-bias-free multi-channel information of the neural activity (Murray et al., 2008).

The Monte Carlo cluster-based permutation test (4,000 randomizations) was used for statistical analysis. Every participant's averaged ERN and CRA neural response was utilized for the statistical analysis, and it was tested if the amplitude of the ERN and CRA waveforms are significantly different from each other, or in other words, if the Training-ErrP signal is significantly different from zero. Based on previous knowledge, the ErrP signals have the highest activity around Fz and Cz electrodes; therefore, the statistical analysis was conducted on EEG signals at the Fz electrode location. The Monte Carlo cluster-based permutation test is widely used to address the multiple comparisons problem for electrophysiological data analysis (Maris and Oostenveld, 2007). Fieldtrip toolbox was used to conduct the cluster-based permutation test (Oostenveld et al., 2011).

### Single-Trial Classification

Detection of ERN signals associated with the Training-ErrP in real-time and a single trial is of utmost importance, as the detection of the ERN signals forms the basis of adapting the external robotic assistance level in the proposed ERN-RASRS. Therefore, ERN signals were classified against the CRA and the background EEG activity as a binary classification problem.

#### ERN vs. CRA Classification

The pre-processed EEG data were pre-segmented into epochs ranging from 0 to 500 ms relative to the onset of Figure 3E. As described earlier, the pre-processed single-trial ERN and CRA signals, without any averaging, were used for classification. We had 180 ERN epochs and 275 CRA epochs in total for all participants, which were all pulled together for classification. The epochs were spatially filtered using the xDAWN spatial filtering algorithm (Rivet et al., 2009; Congedo et al., 2013). Five spatial filters corresponding to the highest eigenvalues were selected for each of the two classes. All 455 epochs were spatially filtered using the two-set of five spatial filters, which resulted in ten projections for each epoch. The grand-average ERN and CRA were also spatially filtered with five spatial filters calculated using ERN and CRA epochs, respectively. The resulting ten projections were concatenated with ten projections of each of the 455 epochs (Congedo et al., 2013; Barachant and Congedo, 2014). Subsequently, a covariance matrix of R^20 × 20^ was estimated for each of the 455 epochs, which was further projected to a tangent vector space using the Riemann metric (Barachant et al., 2013). The above procedure allows exploiting not only the spatial structure but also the temporal structure of the ERP signal, which leads to greater discrimination in ERP classes and, consequently, a higher classification accuracy (Barachant and Congedo, 2014). These xDAWN spatial filtering and Riemannian geometry-based steps were performed using the pyRiemann Python package (2021). The resulting feature vectors were normalized using L1 normalization, and subsequently, the dimensionality of the feature vectors was reduced using principal component analysis (PCA) by keeping components that explained 99% of the data variability.

Support vector machine (SVM), with radial basis function (RBF) kernel, was used to classify the ERN and CRA epochs. Before classification, the class weights of both classes were balanced based on the class frequencies in the input data. A 5-fold cross-validation was used to estimate the classification performance. In each fold, the parameters such as xDAWN spatial filter weight matrices and PCA weight matrices were calculated on the training data only, and the validation set was transformed using these weights. Accuracy, the area under the receiver operating characteristics curve (AUROC), and F1-score were used as performance evaluation metrics. A one-tailed Wilcoxon signed-rank test (exact method) was used to assess if the classification accuracy is significantly higher than the chance level accuracy calculated using the Zero Rule classifier. Zero Rule classifier always outputs the class that is in the majority in the training set, regardless of the input.

#### ERN vs. Background EEG Classification

**Figure 4:**
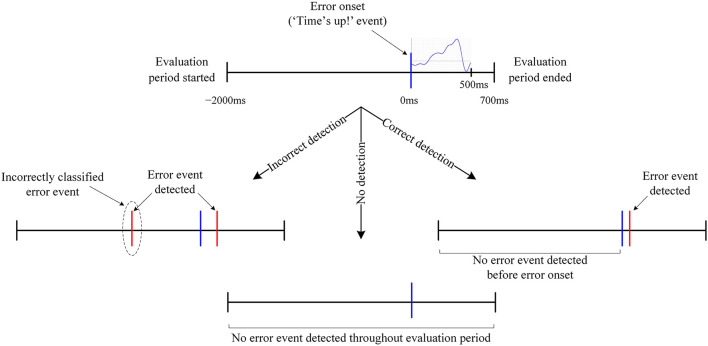
Graphical representation of an error trial structure and the evaluation period. All occurrences that are neither labeled nor detailed inherit their description from the corresponding preceding node. Notably, in no detection, no error event was detected in the evaluation period despite the presence of an error event.

In the asynchronous conduct of the current experiment, the “Time's up” event, such as in Figure 3E, would be unavailable to process the EEG signals synchronously. As a result, the EEG signals cannot be converted to small epochs as done in *ERN vs. CRA classification* for classification. Therefore, the proposed ERN-RASRS requires detecting ERN signals against the background EEG activity in real-time and in a single trial. Here, the feature extraction steps followed were the same as the ones followed in *ERN vs. CRA classification*. The [−2,000, 700] ms interval relative to the error onset (see Figure 4) was used for evaluation. The error onset here refers to the onset of the ERN signal; thus, we had 180 evaluation blocks corresponding to the 180 ERN events.

The SVM classifier, the same as the one used in *ERN vs. CRA classification*, was used for the pseudo-online classification of the ERN signals. The classification was conducted asynchronously by sliding a 500 ms window over the entire evaluation block, with a 20 ms leap in consecutive windows. The classification evaluation of each window resulted if the analyzed window belongs to the ERN class or the background EEG activity class. Thus, the classifier produced an output every 20 ms in the entire evaluation block. We defined an *Error event* detection when an ERN class was predicted for *n* consecutive windows, where *n* is defined as the sensitivity level of the detection algorithm. The detection performance was tested for *n* = {1, 2, 3}.

An error trial was considered correctly classified when no *Error event* was detected before error onset and at least one *Error event* was detected within 200 ms of error onset (see Figure 4 for details). A leave-one-out cross-validation was used to evaluate the classifier's performance. Therefore, cyclically, the evaluation block corresponding to all but one ERN event formed the training set, and the one block left formed the validation set. Notably, there are three classification output possibilities: correct detection, incorrect detection, and no detection (see Figure 4 for details). Correct detection is certainly better than incorrect/no detection, and no detection can be considered better than incorrect detection, as an incorrect detection would result in unwarranted external assistance. However, not a single evaluation metric captures all three output possibilities and gives a single quantitative measure to assess ERN vs. background EEG classification performance. Therefore, to consider all three output possibilities and their significances, we devised a new performance evaluation metric, which is the incidence-detection rate (IDR), to quantify the classifier's performance. The IDR is defined as:


IDR = CD×(1−ID+log(1+ND))

where *CD* is the ratio of correct detection trials, *ID* is the ratio of incorrect detection trials, and *ND* is the ratio of no detection trials to the total number of trials. When all trials are correctly classified, the IDR would be 100%, whereas, when none of the trials is correctly classified, the IDR would be 0%, and for all other values, IDR would vary between 0 and 100%. The whole leave-one-out cross-validation was repeated over 100 times using the bootstrap method, and the upper and lower limit of 99% confidence interval (CI) was estimated. For bootstrapping, after selecting an epoch for validation, the training data epochs were randomly sampled with replacement from the rest of 179 epochs, which were repeated over 100 times.

## Results

### Event-Related EEG Activity

**Figure 5:**
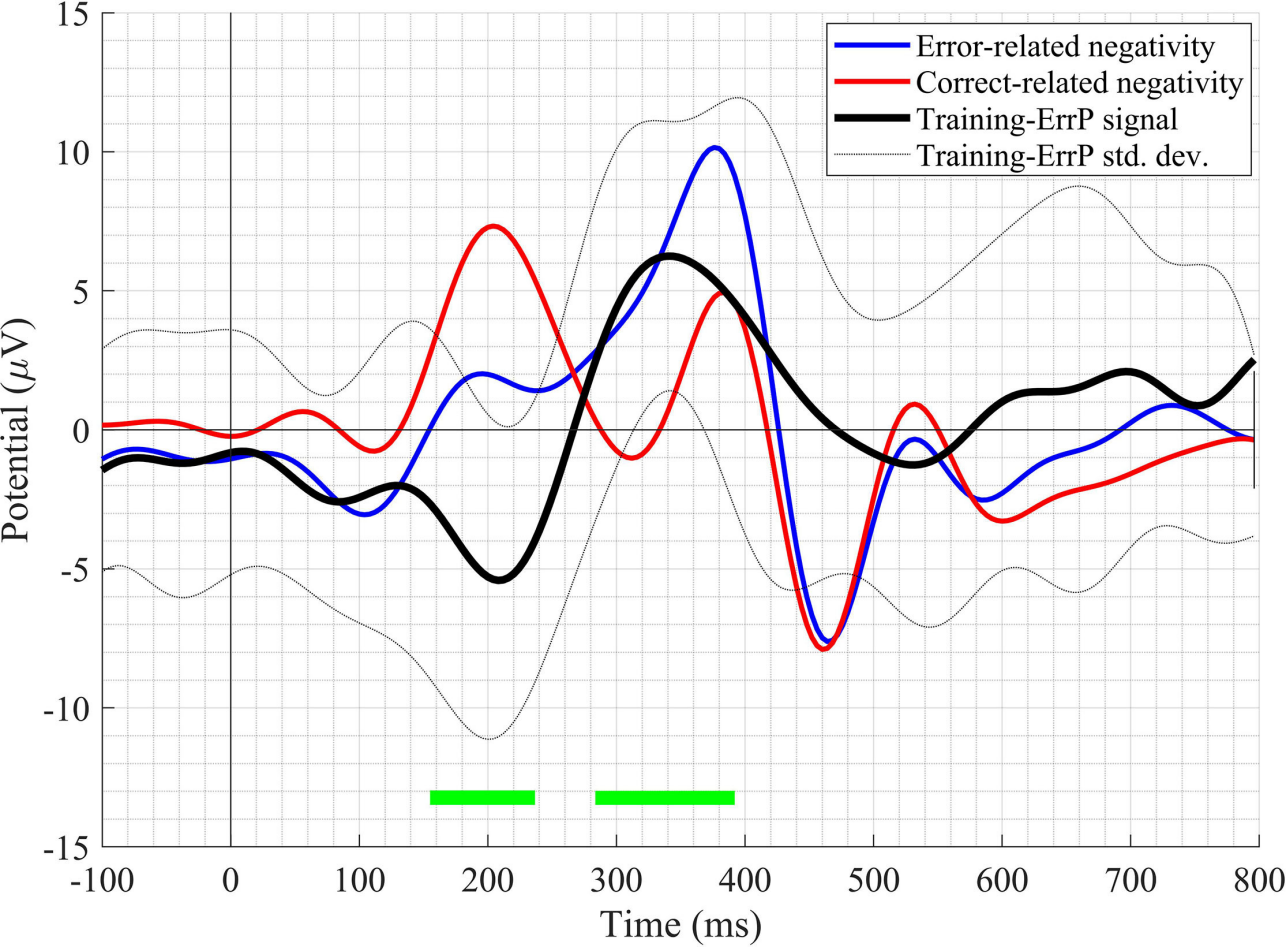
The grand-average waveform of error-related negativity, correct-related activity, and Training-ErrP signal at the Fz electrode location. The green-bars mark the time-points when the Training-ErrP signal is statistically significantly different against zero after cluster-based multiple comparisons correction. For illustration purposes, the waveforms have been smoothed out with a 10 Hz low-pass finite impulse response (FIR) filter.

**Figure 6:**
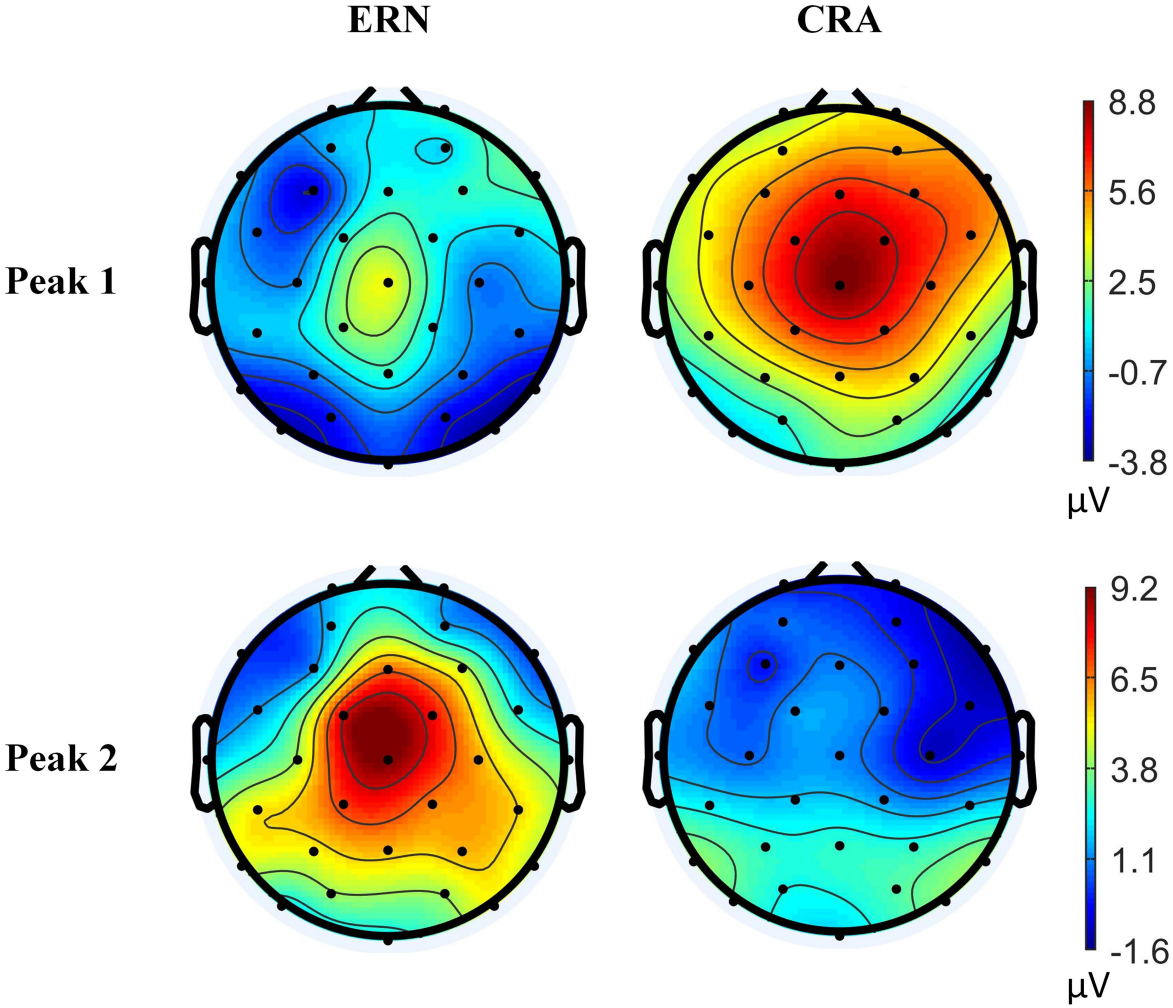
Topographical scalp maps of the grand-average error-related negativity and correct-related activity signals at two time-points, where the Training-ErrP signal was observed to be prominent.

A clear difference in the ERN and CRA neural responses was observed. The grand-average ERN and CRA responses, along with the grand-average Training-ErrP signal at the Fz electrode location, are shown in Figure 5. The Training-ErrP signal has two prominent peaks: a negative peak (peak-1) at 208 ms (*M* = −5.9, *SD* = 6.1), followed by a positive peak (peak-2) at 340 ms (*M* = 5.7, *SD* = 4.2). Both the peaks were observed to be statistically significant (*p* < 0.05) after correcting for multiple comparisons (see Figure 5). The topographical scalp maps also showed a clear and distinguishable neural response between the ERN and CRA (see Figure 6). The ERN signals were observed to be centrally distributed at both peaks. The CRN signals were observed to have been centrally distributed at peak-1, whereas the peak-2 was observed to have a center-frontal distribution.

### Single-Trial Classification

**Figure 7:**
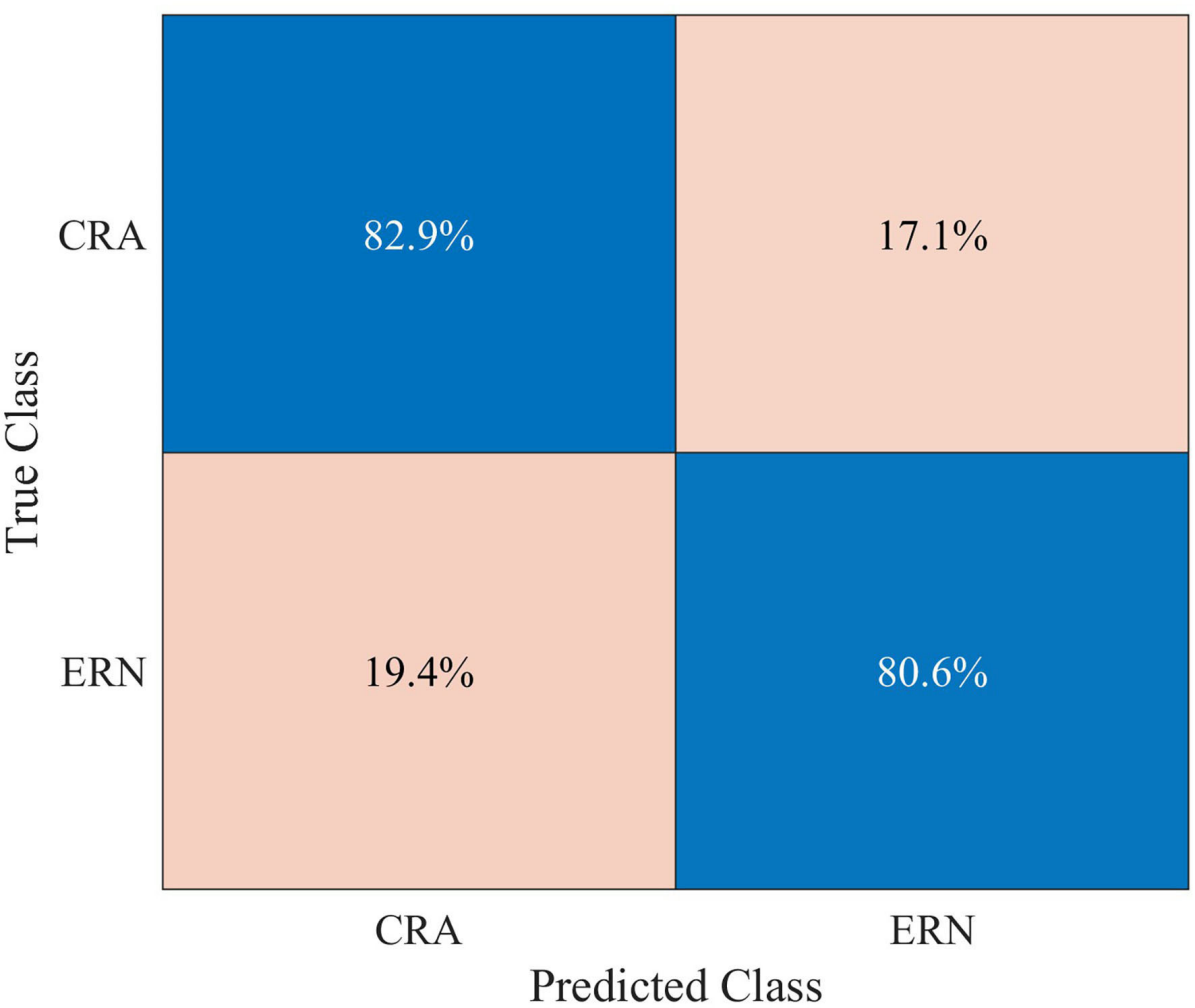
Row-normalized confusion matrix for single-trial binary classification of error-related negativity (ERN) epochs against the correct-related activity (CRA) epochs, using 5-fold cross-validation.

A mean classification accuracy of 0.820 (*SD* = 0.026), AUROC of 0.818 (*SD* = 0.029), and F1-score of 0.821 (*SD* = 0.025) were observed in ERN vs. CRA binary classification, using 5-fold cross-validation. The classification accuracy was significantly higher than the chance level accuracy (*Z* = −2.041, *p* = 0.031), which was 0.604 and was obtained using the Zero Rule classifier. The row-normalized confusion matrix of the ERA vs. CRA binary classification is shown in Figure 7. The participant-wise ERA vs. CRA binary classification results are provided in Supplementary Table 1.

**Figure 8:**
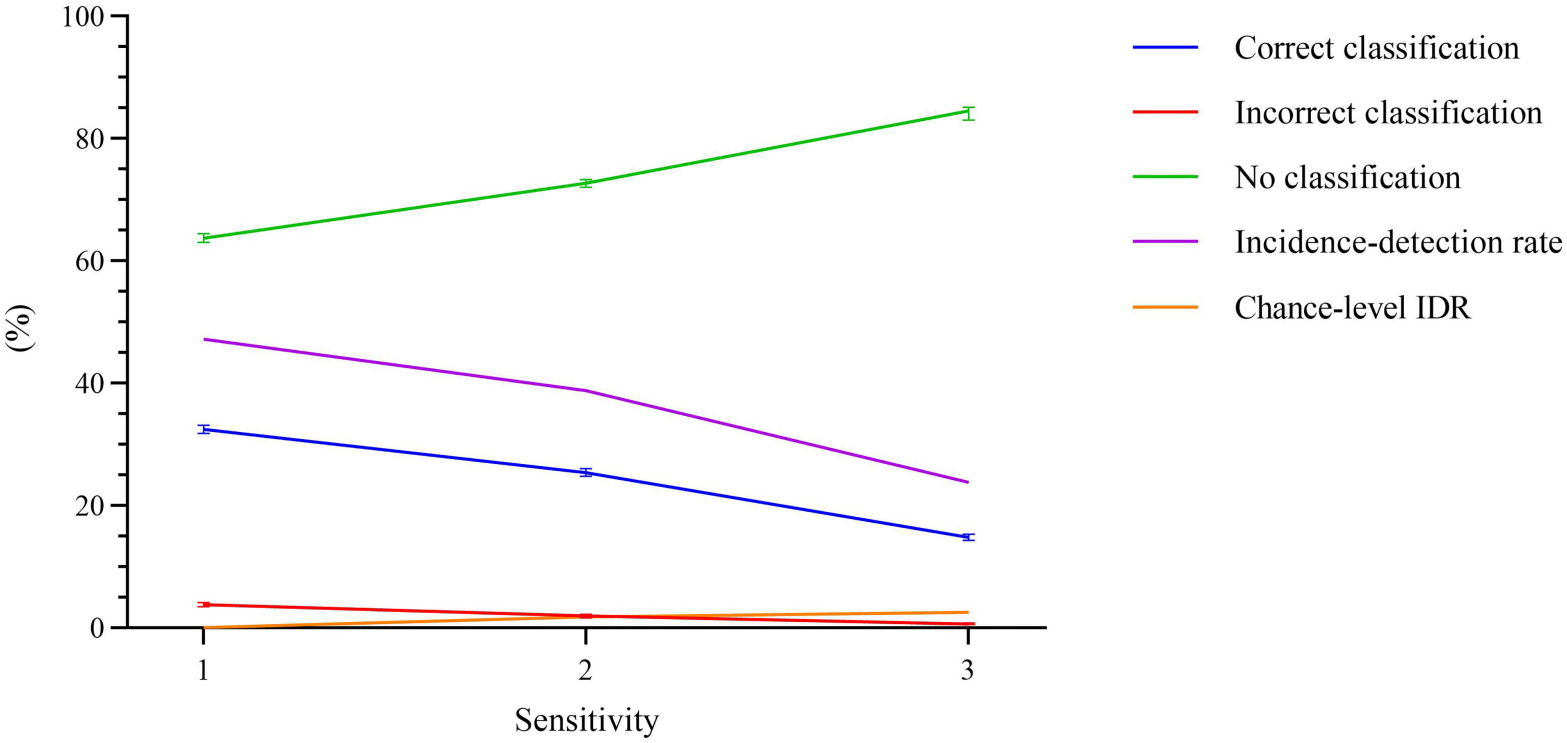
Percentage of error trials that were correctly detected, incorrectly detected, or no detection was observed, as a function of the sensitivity. The error bars represent the 99% confidence interval for the averages. The incidence-detection rate (IDR) metric, represented using pink line, is highest at sensitivity level 1. The chance-level IDR, calculated using a permutation test, is also shown in orange.

The ERN vs. background EEG classification results are shown in Figure 8. The figure depicts the average number of error trials that were correctly detected, incorrectly detected, or no detection was observed, in percentage terms, for each of the three sensitivity levels. The error bars show the 99% CI for the averages. The IDR values were calculated using the average values. The chance-level IDRs were also calculated using a permutation test and are shown in Figure 8. The sensitivity level that maximized the IDR was *n* = 1. Using this level, we obtained an IDR of 47.23%, an average correct classification rate of 32.46%, 99% CI [31.83 33.09], a no classification rate of 63.73%, 99% CI [63.05 64.42], and an incorrect classification rate of 3.81%, 99% CI [3.45 4.16]. The correct classification rate is more than 8.5 times the incorrect classification rate. The participant-wise ERN vs. background EEG classification results are provided in Supplementary Table 2.

## Discussion

The study aimed to propose an ERN-based RASRS. To this aim, the setup of the proposed rehabilitation system was presented, and the working mechanism of the setup was illustrated through a timing diagram. For developing this system, it is imperative to have a distinguishable neural response when a participant fails in performing a rehabilitation exercise against when the participant succeeds and detect these neural responses in a single trial, especially against background EEG activity. Our results showed a statistically significant difference in the brain's neural response when the participants failed in completing the rehabilitation exercise in a given time against when they succeeded. The results showed a significantly higher classification accuracy than the chance level accuracy when classifying ERN signals against CRA signals. When classifying ERN signals against background EEG activity, the correct detection rate of error trials was more than 8.5 times that of the incorrect detection rate. Therefore, the crucial requirements for the proposed ERN-RASRS have been demonstrated.

Range of motion (ROM), voluntary physical efforts, and cognitive investment all play an important role in the rehabilitation but are rarely considered together. Rehabilitation therapies, such as constraint-induced movement therapy (CIMT), focus on voluntary physical efforts in a limited ROM (Abo et al., 2014), whereas passive rehabilitation therapies focus on the ROM of the impaired limb (Caimmi et al., 2017). The EMG (Song et al., 2013) and force-transducer proportional (Colombo et al., 2007) rehabilitation therapies focus on both ROM and voluntary physical efforts. However, the dynamic relation in EMG and force production (Tang and Rymer, 1981), and the abnormal muscle activations (Canning et al., 2000) after stroke decrease the effectiveness of EMG and force-transducer proportional rehabilitation therapies. Moreover, these therapies tend to become unusable for patients with little to no voluntary movement due to the lack of EMG signals and force in the impaired muscles.

On the other hand, ERN-RASRS promotes the balance between the ROM and the patients' voluntary movements with the impaired limb, and it can be utilized not only by patients with voluntary control of their impaired limb but also by patients with little to no voluntary movement. In ERN-RASRS, the balance between the ROM and the voluntary movements can be adapted to a patient by adjusting the level of external assistance (i.e., the *x* and *x*+*1* level, see Figure 2) and the target of the rehabilitation exercises based on the ERN signals. The fixed level of external assistance (i.e., *x* in Figure 2) can be pre-selected and kept high if a patient has little to no voluntary movement and can be kept low if a patient has voluntary control of their impaired limb, which can be further adjusted trial-by-trial based on ERN signals. This can ensure an optimum level of external assistance and desired level of exercise difficulty for every stroke patient and ultimately achieve active and prolonged participation in the rehabilitation program (Shirzad and Van Der Loos, 2016), where the current rehabilitation therapies are expected to be missing the mark. Also, full-ROM in the exercise without losing the voluntary control of their impaired limb and an enhanced level of control on the rehabilitation program by putting their brain's intrinsic feedback in the rehabilitation control loop can engage patients better in the rehabilitation program and motivate them to practice longer, which can result in better recovery (Warraich and Kleim, 2010; Lohse et al., 2014).

The Training-ErrP signal observed in this study has a considerable difference from the ErrP signal observed in Kumar et al. (2019a), which could be a result of different EEG references employed in the two studies, as explained in Murray et al. (2008). Nevertheless, this study's Training-ErrP signals' morphology is similar to previously reported ErrP signals (Chavarriaga and Millán, 2010; Salazar-Gomez et al., 2017). The ERN and CRA topographical activities have been observed to be distributed around the scalp's central region, which seems to corroborate with the fact that the dorsal anterior cingulate cortex/posterior medial frontal cortex are the primary regions of the brain responsible for our error-processing system (Taylor et al., 2007).

The classification approach used resulted in statistically significant ERN vs. CRA classification accuracy, in line with previously reported studies (Salazar-Gomez et al., 2017; Kumar et al., 2018; Torres et al., 2018). On the other hand, against the background EEG activity, about 1/3 of the ERN trials were correctly classified, and incorrect detection and no detection were observed in nearly 4 and 64% of the trials, respectively. Increasing the *n* to 2 or 3 has reduced the incorrect detection rate; however, the correct detection rate also dropped considerably, which reduced IDR. We explored various measures to increases the share of correctly classified trials, including the use of probability measures to increase the classifier's sensitivity toward the ERN class, which increased the number of correctly classified trials. However, the number of incorrectly classified trials also increased. A possible workaround for this issue is that, instead of relying on the raw timing of event *d* (i.e., when ERN signal is detected, see Figure 2) to modify/trigger the robotic assistance (i.e., an event *i* in Figure 2), we can use the exponential moving average of the raw timings. As the correct detection rate is more than 8.5 times that of the incorrect detection rate, the exponential moving average of the raw timing can converge the modify/trigger timing of the robotic assistance to an optimum time, as per varying disability levels of patients. In case of no detection, the robotic assistance level can remain the same as the preceding trial. Thus, the present results support the development of an ERN-RASRS.

## Limitations and Future Work

This study observed a small detection performance of correctly detecting error trials against the background EEG signals, which can be considered a limitation of the current work. The issue can be mitigated by using exponential moving averages; however, greater accuracy is still required before using such a system outside the lab environment. Detecting brain signals against the background EEG activity is a classic problem due to the low signal-to-noise ratio of EEG signals. Nevertheless, it is expected that the classification performance can be improved by having a larger size of the Training-ErrP dataset and by using deep learning-based methods. In the future, we will further explore the experimental protocol, wherein patients would have greater autonomy to start and stop a rehabilitation exercise. Such protocol would require synchronization between Azure Kinect sensor and EEG data stream and the processing of the recorded data in real-time, which will be explored in the future. This study will form the basis of such an investigation. Notably, any variation in the task in the final RASRS setting can introduce variations in the ERN signal (Iturrate et al., 2013), which can be managed by recalibrating and adapting the ERN classifier to the changes. As previous studies have observed ErrP signals and detected them in a single trial in both healthy as well as the disabled population in a wide range of tasks (see (Kumar et al., 2019b) for review), the Training-ErrP elicitation and detection results presented here and consequently, the ERN-RASRS design can be extended to stroke patients.

## Conclusion

This study proposed an error-related negativity-based robot-assisted stroke rehabilitation system, which uses ERN signals as a basis to increase/decrease the robotic assistance given to a patient to complete a rehabilitation exercise. Including a patient's brain in the control loop of a rehabilitation system is quite marked as it allows the use of the patient's intrinsic feedback mechanism for quick adaptation of the rehabilitation system without the presence of a rehabilitation doctor or therapist, which enables patients to carry out their rehabilitation treatment in an unsupervised environment. The present study has reported, for the first time, the design of the ERN-based RASRS and illustrated its working mechanism through a timing diagram. The study has further shown a distinguishable neural response when a participant fails to perform a rehabilitation exercise against when succeeds in a given time. The study has also demonstrated that these neural signals can be detected in a single trial, particularly against background EEG activity, which is crucial for developing the proposed rehabilitation system. In the future, after working on this studies' limitations, we will fabricate the ERN-RASRS and assess its efficacy against established rehabilitation therapies.

## Data Availability Statement

The raw data supporting the conclusions of this article are available upon reasonable request from the authors.

## Ethics Statement

The studies involving human participants were reviewed and approved by the Ethics Committees of the 2nd Hospital of Jiaxing and the First Affiliated Hospital of Shantou University Medical College, China. The patients/participants provided their written informed consent to participate in this study.

## Author Contributions

AK contributed to the idea formation, experiment design, data collection and analysis, result, discussion, and manuscript preparation. LG and JL contributed to ethics approval, participant recruitment, and data collection. JM and SM contributed to results, discussion, and manuscript preparation. JF and XG contributed to ethics approval and discussion. QF contributed to idea formation, experiment design, discussion, and manuscript preparation. All authors contributed to the article and approved the submitted version.

## Funding

This work was supported by the Li Ka Shing Foundation [Grant No. 2020LKSFG03C].

## Conflict of Interest

JM is employed by OMRON SINIC X Corporation, Tokyo, Japan. The remaining authors declare that the research was conducted in the absence of any commercial or financial relationships that could be construed as a potential conflict of interest.

## Publisher's Note

All claims expressed in this article are solely those of the authors and do not necessarily represent those of their affiliated organizations, or those of the publisher, the editors and the reviewers. Any product that may be evaluated in this article, or claim that may be made by its manufacturer, is not guaranteed or endorsed by the publisher.
