# Supplementary material for: Error-Related Negativity-Based Robot-Assisted Stroke Rehabilitation System: Design and Proof-of-Concept
Source: Front Neurorobot. 2022 Apr 25;16:837119. doi: 10.3389/fnbot.2022.837119 (PMC9085417; doi:10.3389/fnbot.2022.837119)
Supplement: Supplementary file 1 [file Data_Sheet_1.pdf]

Supplementary Materials

Table S1. Single-trial binary classification of error-related negativity (ERN) epochs against the correct-related activity (CRA) epochs for each patient. The detection rate percentages are row-normalized.

Patient	Normalized detection rate of ERN (%)	Normalized detection rate of CRA (%)
1	70.59	100.00
2	88.89	100.00
4	88.89	68.00
5	77.78	80.77
6	80.00	96.15
7	78.57	76.92
8	88.89	92.31
9	81.25	70.83
10	64.29	82.61
11	71.43	90.91
12	77.78	92.00

Table S2. Percentage of error trials that were correctly detected, incorrectly detected, or no detection was observed for each patient against background EEG activity. The detection rates were calculated for sensitivity level 1. The Incidence-detection rate (IDR) for each patient is also shown.

**Patient**	**Correct classification (%)**	**Incorrect classification (%)**	**No classification (%)**	**IDR (%)**
1	15.18	2.47	82.35	23.92
2	29.94	1.06	69.00	45.34
4	54.67	4.67	40.67	70.77
5	32.67	5.22	62.11	46.74
6	15.53	2.60	81.87	24.42
7	16.43	5.21	78.36	25.08
8	52.22	6.28	41.50	67.07
9	45.63	2.75	51.63	63.36
10	43.57	5.64	50.79	59.01
11	14.36	3.86	81.79	22.38
12	29.44	2.44	68.11	44.02
